# Application of Artificial Intelligence in Emergency Nursing of Patients with Chronic Obstructive Pulmonary Disease

**DOI:** 10.1155/2021/6423398

**Published:** 2021-11-24

**Authors:** Lingzhi Hong, Xufang Cheng, Deming Zheng

**Affiliations:** ^1^Department of Emergency, The First People's Hospital of Chun'an County, Hangzhou, Zhejiang 311700, China; ^2^Department of Nursing, The First People's Hospital of Chun'an County, Hangzhou, Zhejiang 311700, China; ^3^Department of Infection, The First People's Hospital of Chun'an County, Hangzhou, Zhejiang 311700, China

## Abstract

The research achievements of artificial intelligence technology in the development of chronic obstructive pulmonary disease were explored, and the advantages and problems encountered in the development of intelligent nursing were analyzed. This paper presents the application of artificial intelligence in the emergency care of patients with chronic obstructive pulmonary disease. The method included 447 COPD patients in a randomized controlled trial to observe the improvement of quality of life at 4 and 12 months after artificial intelligence medical intervention. A prospective randomized controlled trial included 101 patients with COPD who underwent a 9-month web-based knowledge exercise on the prevention of acute exacerbation of COPD through artificial intelligence medicine and were randomly divided into two groups: the experimental group and the control group. The results show that, in the experimental group and the control group, after 4 months, the quality of life does not change; after 12 months, compared with controls, the quality of life and emotional and psychological conditions have improved obviously. 29 patients who participated in the experiment and were randomly divided into the experimental group and the control group showed satisfactory results. COPD hospitalized rate and length of hospital stay were decreased in the experimental group than in the control group. For single-factor analysis, artificial intelligence medical intervention has not achieved significant significance, and the experimental results have preliminarily confirmed the effectiveness of artificial intelligence medical treatment.

## 1. Introduction

In recent years, there are many clinical research reports on the application of artificial intelligence in medical tracking and management, mainly including the changes of pulmonary function and quality of life, the number of deterioration, hospitalization rate, mortality, and patient satisfaction. These studies have achieved some results. After optimizing the allocation of medical resources, they provide patients with better medical services [1]. The application of Internet of Things in AICOPD mainly includes long-term monitoring and management, rehabilitation training, and self-management. Among them, the monitoring and management technology is relatively mature, mainly including computer, mobile phone, network, and telephone as well as real-time transmission of patients' condition changes, which is conducive to the timely intervention of medical workers and improves the medical intelligence. More and more attention has been paid to COPD patients, and the efficacy has been fully affirmed [2]. Pulmonary rehabilitation is based on the overall assessment of tailor-made comprehensive intervention for patients, including sports training, education, and self-management, which aims to improve the pulmonary ventilation function, relieves or controls AICOPDAI acute symptoms and complications and psychological dysfunction, eliminates the disease remains, and helps patients get better quality of life. Artificial intelligence medical treatment for lung rehabilitation training is more convenient and easy, greatly reducing the economic burden of patients [3]. A feasibility study showed that patients with severe AICOPDAI received lung rehabilitation training and psychological counseling for AI3AI week through home remote video mode, under the guidance of specialized rehabilitation therapists. The results showed that 74% of patients with AI3AI completed the study, which was highly popular [4]. AICOPDAI clinical questionnaire, 5AI sitting test, and time walking test were significantly improved in patients who completed rehabilitation training compared with those before rehabilitation training, and the acute exacerbation rate and mortality rate were reduced [5]. Some scholars have reported that the possible reason is that rehabilitation training beyond AI7AI days can improve respiratory muscle function and ventilation of patients. However, the popularity and patient completion rate decreased significantly, which may be related to the poor tolerance of lung function in patients with severe COPDAI. In addition, a series of meta-analyses and clinical reviews have reported that telerehabilitation training can reduce the readmission rate and mortality of patients with varying degrees of AICOPDAI, and the cost of telerehabilitation training is significantly lower than that of traditional pulmonary rehabilitation training. However, because of insufficient evidence, telerehabilitation is not recommended for patients with acute exacerbations or after hospitalization for acute exacerbations. At present, the traditional early prevention and screening of AICOPDAI mainly include risk factors, imaging, lung function, respiratory questionnaire score, 6 min AI walking test, molecular markers, and special genes. The main method is outpatient treatment, which has low efficiency [6] and limited resources, and most patients lack careful and detailed consultation and guidance [7]. Artificial intelligence medical care helps to simplify and improve the current situation, mainly through the corresponding mobile client input required data, such as risk factors, lung function, imaging, electrocardiogram, dyspnea index, CATAI score, diagnosis and treatment guidelines and popular science information, and screening and prevention of AICOPDAI population [8]. For patients with difficulty in diagnosis and treatment, the Internet of Things technology can even be used for remote consultation or multidisciplinary consultation, and professional advice can be given by authoritative people [9]. To solve this research problem, Li et al. developed an APP in the computer, GaitTrack, to monitor the health status of patients by monitoring their walking patterns and accurately distinguish between COPD patients and non-COPD patients. Self-recognition diagnosis for early COPD patients in community services provides a simple and effective model [10]. Hadi and Rigoberto monitoring system can design the mobile signs automatic acquisition of the patient's pulse, blood pressure, ECG, blood oxygen, and other vital signs' information and provide electronic health records in the database to the community resident service platform every day. The paramedics can safely access the patient's condition and timely warning, possible complications, and early detection of patients with COPD; this reduces the workload of community nursing staff and is conducive to the daily monitoring and nursing of COPD patients to ensure that patients can receive adequate professional care in time [11]. Belén et al. proposed that the acute exacerbation warning mode can detect the possible acute exacerbation events related to COPD through the signal of acute exacerbation obtained by using the *in vitro* monitoring equipment and timely inform patients and their families of the critical situation, which can buy enough time for patients in the event of accidents. Real-time warning and effective dynamic management of adverse events are realized [12]. Therefore, the *in vitro* monitoring equipment can play a positive role in the management of patient information by medical staff and self-management by patients [13]. Artificial intelligence (AI) combined with care can help nurses to regulate content of assessment, comprehensive and accurate patient information collection, and exercise the clinical thinking ability quickly, and nursing diagnosis, timely and accurate comprehensive engineering implementation, and nursing quality can be traced all the way, which in turn reduced the incidence of adverse nursing major events and improved patient satisfaction. However, smart systems tend to collect more user data in order to provide better comprehensive care, but the more the private data are collected, the higher the risk of privacy leakage. For example, remote monitoring of vital signs is an effective and timely way to understand patient care needs, which has important clinical significance. However, once such information is maliciously used and it opens a convenient door for criminals. In particular, smart devices are often placed in a patient's private space, such as the bedroom, which means more data are private and further increases the risk of leakage. Based on current research, this paper proposes the application of artificial intelligence in the emergency care of patients with chronic obstructive pulmonary disease. This method included 447 COPD patients in a randomized controlled trial to observe the improvement of quality of life at 4 and 12 months after artificial intelligence medical intervention. A prospective randomized controlled trial included 101 patients with COPD who underwent a 9-month web-based knowledge exercise on the prevention of acute exacerbation of COPD through artificial intelligence medicine and were randomly divided into two groups: the experimental group and the control group. The results show that, in the experimental group and the control group, after 4 months, the quality of life does not change; after 12 months, compared with controls, the quality of life and emotional and psychological conditions have improved obviously. 29 patients who participated in the experiment and were randomly divided into the experimental group and the control group showed satisfactory results. COPD hospitalized rate and length of hospital stay were decreased in the experimental group than in the control group. The single-factor analysis of AI medical intervention has not achieved significant significance, and the experimental results have preliminarily confirmed the effectiveness of AI medical treatment [14].

## 2. Methods

### 2.1. The Necessity of Artificial Intelligence in the Nursing of COPD Patients

Artificial intelligence refers to intelligence implemented by computers. Its ultimate goal is to create intelligent mechanical devices that deal with data according to human thought patterns, so that they can replace human beings in some tasks. In modern medical work, patient-related health data are large in scale and complex in type and needs timely processing and value refinement. This feature of big data means not only a multiplication of workload for medical staff but also a multiplication of diagnosis and decision-making errors [15]. The way to manage these data effectively is using AI. Research shows that nursing staff can improve their work efficiency by applying AI-assisted medical services to timely deal with the care problems of a large number of patients. In recent years, the application of artificial intelligence in the nursing of COPD patients has become more and more prominent. First, AI is able to track and document COPD patients over time. Because the treatment of COPD includes drug therapy, education, self-management, physical activity, sports training, oxygen therapy, and other long-term and regular comprehensive treatment and nursing, nurses need to do a good job of continuity of care for patients, which requires a large number of patient data that need to be evaluated and processed. Under the current basic national conditions in China, the number of patients and long-term data far exceed the working intensity that medical personnel can bear, while AI's powerful data analysis and recording function can meet the needs of continuous care of COPD patients, help nursing staff solve the huge amount of information, and reduce the task [16]. Second, AI is able to personalize care for COPD patients based on their condition. COPD is a chronic disease that is often characterized by progressive airflow restriction. Moreover, most COPD patients do not pay enough attention to their own disease, and their treatment compliance is poor. They cannot strictly follow the doctor's advice for treatment or rely on the supervision of medical staff. This presents a huge challenge for nurses to adjust nursing goals based on patient compliance and treatment outcomes during stable COPD. At the same time, the pulmonary rehabilitation of COPD patients requires nurses to judge patients' exercise intensity according to the degree of airflow restriction, which means that nurses have to spend more time as supervisors and companions, which are difficult to be fully realized in the current era of nursing personnel resource shortage. The appearance of AI can detect the body condition of COPD patients at anytime and anywhere, instead of nurses to determine the most suitable exercise program and exercise intensity of patients and make corresponding adjustments. Therefore, AI can well fit the uniqueness of COPD patient care and promote the development of the information-based nursing model for COPD patients [17].

### 2.2. Randomized Controlled Experiment

Data 1: a randomized controlled trial of 447 patients with COPD was conducted to observe the improvement in quality of life at 4 and 12 months after artificial intelligence medical intervention.

Data 2: a prospective randomized controlled trial included 101 patients with COPD who underwent a 9-month web-based knowledge exercise for the prevention of COPD exacerbations through artificial intelligence medicine and were randomly assigned to the telemedicine treatment group and control group.

## 3. Results and Analysis

### 3.1. The Results

**Figure 1:**
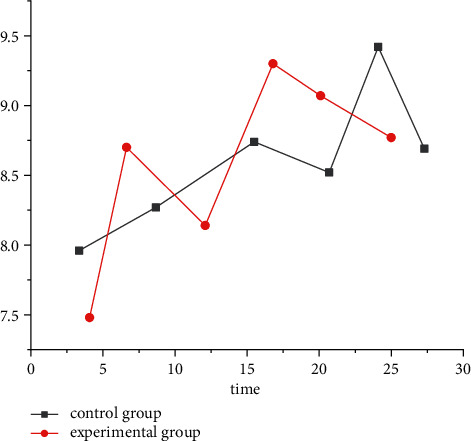
The quality of life in the experimental group and the control group after 4 months.

**Figure 2:**
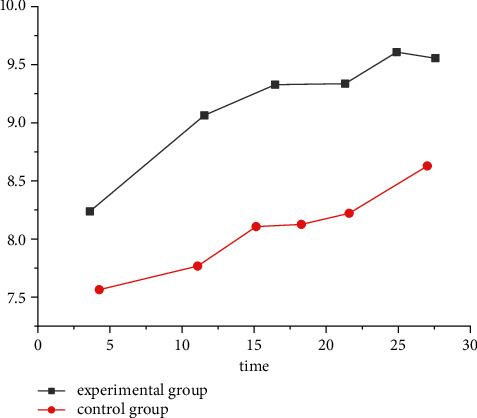
The quality of life in the experimental group and the control group after 12 months.

The improvement of quality of life at 4 and 12 months after artificial intelligence medical intervention was observed. The results showed that there was no change in quality of life in the experimental group and the control group after 4 months, as shown in Figure 1. After 12 months, the quality of life of the experimental group significantly improved compared with the control group, as well as the emotional and psychological conditions, as shown in Figure 2. However, these effects were mainly significant in patients away from acute aggravating risk factors, while the single-factor analysis of AI medical intervention did not achieve significant significance.

**Figure 3:**
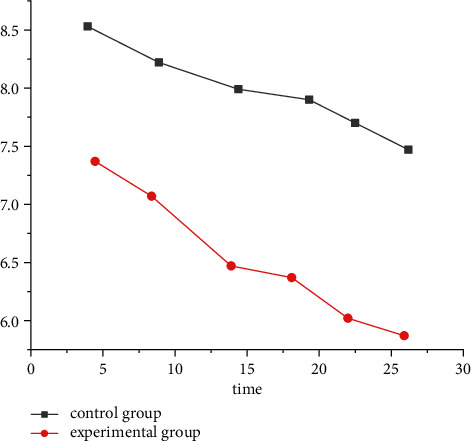
Comparison of COPD hospitalization rate and length of stay in the experimental group and the control group.

29 patients who participated in the experiment and were randomly divided into the experimental group and the control group showed satisfactory results. The hospitalization rate and length of stay in the experimental group were lower than those in the control group, see Figure 3. The intervention measures in the experimental group were satisfied with the patients and promoted. Although 72 COPD patients withdrew from the clinical trial due to dissatisfaction with the evaluation criteria of Internet problems after the beginning of the study, as shown in Table 1, the test results preliminarily confirmed the effectiveness of AI medical treatment [18].

### 3.2. AI Technologies Related to the Care of COPD Patients

At present, the application of artificial intelligence in COPD nursing is mainly in community service, patient follow-up, home nursing, rehabilitation training, and so on, and the equipment's main goal is to abate patients outside the court without professional medical staff because of the lack of early prevention and self-management ability that lead to the risk of acute episodes and to reduce hospital readmission rate and mortality of patients. In view of the difficulties in long-term effective supervision and management of COPD and complicated data processing of medical staff, many artificial intelligence nursing technologies have been introduced in the field of AI, mainly including smart phones, *in vitro* monitoring equipment, intelligent robots, and virtual reality technology. The occurrence of these technical equipment commodities has great enlightenment and practical significance for the nursing form of COPD patients under the background of intelligent big data analysis [19].

#### 3.2.1. Smart Phones

Smart phone, as one of the important carriers of mobile health technology, can be convenient and effective to fund COPD patients to retain and transmit their own disease data, and at the same time, it also facilitates the analysis and sorting of data by nurses and effective communication with COPD patients. Smart phone applications have been widely developed to play a positive role in COPD self-management, health promotion, and treatment compliance. Smart phone, as a basic carrier of artificial intelligence, can be combined with *in vitro* monitoring equipment, robot, and virtual reality technology to assist health and medical work; there is more space for development in the future.

#### 3.2.2. *In Vitro* Monitoring Equipment

On the basis of convenient data analysis of mobile phone software, some developers will be equipped with wearable *in vitro* monitoring equipment, convenient for patients and medical staff to see the physical condition report anytime and anywhere, effectively self-management. Especially when patients are out of hospital, *in vitro* monitoring equipment can be a good substitute for the medical staff plan to evaluate patients. The first is the community. With the monitoring of intelligent mobile terminals and wearable devices, the health status of some first-time patients can be assessed in advance. As a result, this AI technology can help the COPD population to conduct scientific preliminary analysis and evaluation, and at the same time, healthcare workers can use chronic disease community management systems and *in vitro* monitoring equipment to obtain and manage patient information for the entire community. From many aspects of social group characteristics and personal characteristics with the help of intelligent in-depth analysis, classification and refinement of chronic disease prevention, treatment, nursing, and rehabilitation needs, AI improves the professional level of nursing staff. Thus, community can set up electronic health records for every resident and conduct big data analysis of health information through cloud application platform. Combined with in vitro monitoring equipment, it can help nurses master the patient's health information data and realize effective nursing evaluation and diagnosis. At the same time, many remote home self-management system platforms have been designed by researchers aiming at the poor treatment compliance of COPD patients at home. If a study allows patients to equip themselves with a simple pulse oximeter and a small gas volume monitor at home, the measurement results (their own pulse rate, blood oxygen saturation, and forced expiratory volume in 1 second) can be directly transmitted to the self-management system platform through Bluetooth. The instrument analyzes and compares the results of the patient's analysis through the probabilistic model and transmits the results to the service center through the network health data transmission system.

#### 3.2.3. Intelligent Robots

In order to improve the quality of life of COPD patients and promote physical and mental health, intelligent nursing robots with different functions have gradually been applied to COPD patients' daily life, entertainment life, disease management, and physical rehabilitation. In some foreign studies, diet nursing robot, companion robot, rehabilitation assistance robot, and remote health management robot have been applied in COPD patients. A robot decision support system (DSS) can be used to automatically monitor and control COPD patients' physical exercise training, so that patients can do rehabilitation exercise in the community home. This decision-making system can replace nurses to timely help and correct patients' rehabilitation exercise, complete the basic needs of COPD patients in life and necessary auxiliary treatment, and effectively manage patients' disease changes in the long term. The upper limb rehabilitation robot was used to provide different rehabilitation modes and exercises of different intensities according to the physical condition of COPD patients. The results showed that the rehabilitation effect was positively evaluated by patients and physiotherapists. They can provide personalized care for patients with COPD and help patients with stable disease to better cooperate with respiratory exercise at home [20]. Thus, the emergence of intelligent robots can not only improve the quality of life of patients and comprehensive care of patients' physical and mental health but also reduce the physical contact of medical staff to patients and at the same time can effectively reduce the risk of COPD patients infected with infectious diseases. On the other hand, the use of robots to help COPD patients independently or to assist to complete rehabilitation exercise can save a lot of energy and workload of nurses, effectively improve the utilization rate of existing human resources, and relieve the situation of nursing human resources tension.

## 4. Conclusions

This paper proposes the application of artificial intelligence in the emergency nursing of patients with chronic obstructive pulmonary disease. The method included 447 patients with COPD in a randomized controlled trial that looked at improvements in quality of life at 4 and 12 months after AI medical intervention. A prospective randomized controlled trial included 101 patients with COPD who underwent a 9-month web-based knowledge exercise on the prevention of acute exacerbation of COPD through artificial intelligence medicine and were randomly divided into two groups: the experimental group and the control group. The results show that the quality of life of the experimental group and the control group has no change after 4 months and the quality of life of the experimental group significantly improved compared with the control group after 12 months, and the emotional and psychological conditions also significantly improved. Twenty-nine patients who participated in the trial and were randomly divided into the telemedicine experimental group and the control group showed satisfactory results, with lower COPD hospitalization rates and length of stay in the experimental group compared with the control group. For single-factor analysis, artificial intelligence medical intervention has not achieved significant significance, and the experimental results have preliminarily confirmed the effectiveness of artificial intelligence medical treatment. With the steady development of COPDAI technology equipment and the correct guidance of government departments, COPDAI has attracted more and more attention in the field of medical care. In the future, the mode of medical care service will change dramatically and the application prospect is broad. On the one hand, COPDAI technology can provide better nursing services for patients and improve the rehabilitation effect and quality of life of patients. On the other hand, COPDAI technology will involve ethical issues and technical difficulties in patients' privacy and requires the multidisciplinary collaboration of medical staff and computer experts to continuously develop applications more suitable for patients' conditions. Nurses need to change the traditional nursing mode and fully apply COPDAI as a basic medical tool to the nursing work of families, communities, and hospitals. At the same time, we should pay attention to meet the needs of patients as much as possible, respect the wishes of patients, and provide ideas for improving research and development.

## Figures and Tables

**Table 1:** Patients' satisfaction with Internet problems.

Group	Number of cases	Satisfaction	Dissatisfaction
Men	51	19	32
Women	50	10	40

## Data Availability

The data used to support the findings of this study are available from the corresponding author upon request.
